# Evidence of West Nile Virus Infections in Wild Boars (*Sus scrofa*) in the Netherlands, 2018–2021

**DOI:** 10.1111/zph.70088

**Published:** 2026-09-06

**Authors:** Kiki Streng, Eduardo de Freitas Costa, Sophie van Oort, Renate Hakze‐van der Honing, Gebbiena Bron, Carl Andreas Grøntvedt, Wim H. M. van der Poel, Nathanaël Hozé, Michel Counotte

**Affiliations:** ^1^ Infectious Disease Epidemiology Wageningen University & Research Wageningen the Netherlands; ^2^ Epidemiology, Bio‐Informatics & Animal Models Wageningen Bioveterinary Research Lelystad the Netherlands; ^3^ Virology & Molecular Biology Wageningen Bioveterinary Research Lelystad the Netherlands; ^4^ Animal Health, Animal Welfare and Food Safety Norwegian Veterinary Institute Ås Norway; ^5^ Université Paris Cité and Université Sorbonne Paris Nord IAME, INSERM Paris France; ^6^ Institut Pasteur Epidemiology and Modelling of Antimicrobials Evasion Research Unit Paris France; ^7^ Université Paris‐Saclay UVSQ, Inserm, CESP, Anti‐Infective Evasion and Pharmacoepidemiology Research Team Montigny‐Le‐Bretonneux France

**Keywords:** Bayesian model, orthoflavivirus, serology, surveillance, wild boar

## Abstract

**Introduction:**

Orthoflaviviruses are emerging in Europe and have potential significant impact on animal and public health. Usutu virus (USUV) and West Nile virus (WNV) have been first detected in the Netherlands in 2016 and 2020, respectively. Wild boars (*Sus scrofa*) have been suggested as an interesting population to detect the circulation of orthoflaviviruses. We aim to provide epidemiological insights of USUV and WNV infections in wild boars in the Netherlands. We further explore the interpretation of serological results, taking into account cross‐reactivity patterns.

**Methods:**

A total of 1504 wild boar sera from 2018 to 2021 were tested using a screening ELISA. Positive samples were confirmed using Virus Neutralization Tests. We explain the observed serology of the individuals with a 3‐level Bayesian hierarchical model.

**Results:**

We found evidence for WNV infections in boars throughout the study period, with probabilities of past infections ranging from 7.82% in 2020 up to 13.02% in 2019. Our results show a declining trend in past USUV infections over the four‐year study period from 24.00% in 2018 to 5.37% in 2021. Furthermore, we estimate direct and cross‐reactive titre responses to both viruses, including the probability of developing a cross‐reactive response.

**Conclusions:**

This study indicates that WNV infections in wild boars occurred before the first human cases were detected in 2020. Surveillance of orthoflaviviruses in wild boars could provide a convenient method to monitor mosquito‐borne viruses such as WNV and USUV.

## Impacts

- West Nile virus (WNV) infections were detected in wild boars before the first detection of cases in humans in 2020
- The use of a statistical model to analyse serological data improves our understanding of Usutu virus (USUV) and WNV circulation compared to standard interpretation methods.
- Integrating serological surveillance in wild mammals (such as wild boar) into existing monitoring programs can enhance early detection and risk assessment for USUV and WNV.

## 1 Introduction

Orthoflaviviruses are emerging in Europe and have potential significant impact on animal and public health. West Nile virus (WNV) and Usutu virus (USUV) are viruses from the genus *Orthoflavivirus* in the family *Flaviviridae* (Lefkowitz et al. 2018). Both are transmitted by mainly *Culex* spp. mosquitoes and maintained in an enzootic cycle between (wild) birds and mosquitoes (Nikolay 2015). Infections of these orthoflaviviruses in humans often result in asymptomatic or mild disease. However, WNV infections may cause significant disease and even mortality in a small proportion of infected individuals (ECDC and EFSA 2023). In other mammals, infections often go unnoticed except for WNV infections in horses (WOAH 2018). Both viruses are currently co‐circulating in Europe (Beck et al. 2013). In 2016, USUV was detected in the Netherlands and has been circulating since (Oude Munnink et al. 2020; Rijks et al. 2016). WNV was first detected in 2020 in birds and mosquitoes (Sikkema et al. 2020). That year, eight autochthonous human cases were identified (Vlaskamp et al. 2020). Circulation of WNV was detected by serology in sentinel chickens in 2021 (Streng, Atama, et al. 2024). The virus was detected again in a single bird in 2022 (RIVM 2022) and in mosquitoes in 2025 (RIVM 2025). Furthermore, in 2025, the first official equine WNV cases were identified in the country (RIVM 2025).

As viremia is cleared rapidly, virus detection in dead‐end hosts is often unsuccessful, and thus serological methods must be used. However, co‐circulation of orthoflaviviruses hampers diagnosing infections. Antigenic cross‐reactivity complicates the interpretation of serological studies as WNV and USUV are both part of the Japanese Encephalitis serocomplex (Beck et al. 2013). Tick‐borne encephalitis virus (TBEV), another orthoflavivirus that is also present in Europe, is part of a different serocomplex and antibodies are thus not expected to cross‐react in Virus Neutralization Tests (VNTs). Generally, sera are first screened using an antibody binding approach such as an ELISA or Protein Microarray. Neutralization assays, such as a VNT or Plaque Reduction Neutralization test (PRNT) are regarded as the gold standard to confirm infection (WOAH 2018). A four‐fold difference in neutralizing antibody titers between different orthoflaviviruses is usually regarded as a determining factor for confirmation of past infection (WOAH 2018).

Interpretation of neutralizing antibody titers is further complicated by factors such as individual variation in antibody development, waning of antibodies over time, coinfections, reinfections and others (Beck et al. 2013; Rathore and St. John 2020). This may lead to the inability to distinguish infections in individuals and thereby incorrect prevalence estimations. Diagnostic algorithms may guide interpretation of currently available serological tests (Cavalleri et al. 2022; Piantadosi and Kanjilal 2020). Development of more specific diagnostic tests is another, challenging, area of focus for research. Until these tests exist, other approaches must be explored to deal with serological interpretation of orthoflavivirus infections. One approach is statistical modelling of serological results in populations which explores circulation of multiple pathogens in the same population, considering co‐circulation and cross‐reactivity. Studies using this approach have been published for pathogens like influenza A virus, chikungunya and o'nyong‐nyong virus, and SARS‐CoV‐2 (Glemain et al. 2024; Hozé et al. 2021; Kucharski et al. 2018).

Wild boars ( *Sus scrofa* ) are suggested to be good species for orthoflavivirus surveillance, as they potentially experience high exposure to mosquito bites and live in forest rich areas (Adjadj et al. 2022; Gutiérrez‐Guzmán et al. 2012; Petruccelli et al. 2020). Even though they do not play a role in transmission, detection of (past) infection can indicate (past) circulation even in dead‐end hosts, similar to sentinel chickens (Halliday et al. 2007). The Netherlands maintains an ongoing surveillance program in wild boars to preserve its disease‐free st Fever, African Swine Fever, and other relevant pathogens (Wageningen University and Research 2024).

The study presented here aims to provide epidemiological insights of USUV and WNV infections in wild boars in the Netherlands from 2018 to 2021. We further explore the interpretation of serological results while taking into account cross‐reactivity patterns of these two viruses in wild boars. We provide suggestions on how our findings may be incorporated in current and future surveillance programs.

## 2 Materials and Methods

### 2.1 Dutch Samples (2018–2021)

Wild boar ( *S. scrofa* ) samples used in this study were collected as part of the ongoing surveillance program in wild boars in the Netherlands. Wild boar habitats are widely present throughout mainly the South‐East of the Netherlands and the number of boars has been rising over the past decade. Serum was taken by hunters after shooting (cardiac puncture) and was sent to the laboratory of Wageningen Bioveterinary Research in Lelystad, the Netherlands. Demographic data were provided by hunters via a submission form in most cases. Age (in months) was estimated by assessment of the teeth. The required sample sizes were calculated to detect a seroprevalence of 1% with a confidence level of 95% and a precision of 99% (Sergeant 2018; Thrusfield 2018). A random selection of approximately 380 sera was included for each year of the study period.

### 2.2 Serological Testing and Reference Sample Sets

All samples were first screened for orthoflavivirus antibodies by a commercial species‐independent ELISA according to the manufacturer's protocol (ID Screen, West Nile Competition Multi‐species, IDvet Innovative Diagnostics, France). This ELISA is known to show cross‐reactive results for WNV, USUV, as well as TBEV in animals (Beck et al. 2017). Doubtful and positive sera were subsequently tested in duplicate in Virus Neutralization Tests (VNTs) for WNV and USUV according to a previously described method (Streng, Hakze‐van der Honing, et al. 2024). Sera from an experimentally WNV infected horse and a field‐infected USUV owl were used as positive controls.

Due to the low quality of wild boar serum samples, aspecific reactions in VNTs might occur. Therefore, forty‐one sera with negative ELISA results were randomly selected to be tested in VNTs, to assess the possibility of false positives in VNT, assuming a perfect sensitivity of the ELISA. Two additional sets of negative control populations were used to estimate the VNT cut‐offs. Firstly, slaughterhouse samples of domestic Dutch pigs (*n* = 14) were used. Secondly, a set of sera from Norwegian wild boars (*n* = 50) hunted in 2018 and 2019 were used as negative control population as both USUV and WNV have not been detected in Norway (ECDC 2021). ELISA and VNT results of the Norwegian wild boars and domestic Dutch pigs are shown in Figures S1 and S2. Based on these results, neutralizing titers ≥ 10 were regarded positive for USUV; for WNV, a neutralizing titre of ≥ 30 was regarded positive.

We assumed no cross‐reactivity to TBEV in VNTs, and therefore only WNV and USUV titers were taken to the model. Titers were read as the reciprocal of the highest dilution for which at least 50% cytopathic effect (CPE) was observed. All sera were tested in duplicate, and outcomes of both replicates were registered individually. For the classical interpretation, the mean titre of the two duplicates was calculated and registered as the end titre. A proportion of sera were of very bad quality and could either not be tested or CPE could not be interpreted in one or more VNTs. Therefore, these sera were removed from further analyses and not included in the statistical model.

### 2.3 Statistical Analysis

We explain the observed serology of the individuals in the survey with a model that jointly estimates the probability of infection by WNV and/or USUV at each of the four sampling periods 2018–2021 (*t* = year of sampling), the antibody response upon infection, the impact of cross‐reactivity on the antibody titers and the observation errors.

#### 2.3.1 Hierarchical Modelling

We built a 3‐level Bayesian hierarchical model based on Hozé et al. (2021) (Figure 1), which considers the (1) infection status and (2) the antibody response, either due to a direct response, or cross‐reactivity. We extended the model with another level called (3) the observation model. The observation model takes into account the observational error due to an imperfect diagnostic method in the two replicates. Assuming the infection history ($I_{j}$) and the real titers for WNV and USUV respectively ($r_{j}^{W},r_{j}^{U}$) are known, we write this model as:

$$\begin{array}{c} P\left(\mathrm{Ob}s_{j}^{W},\mathrm{Ob}s_{j}^{U},r_{j}^{W},r_{j}^{U},I_{j}|\theta \right)=P\left(I_{j}\;|\theta \right)\;P\left(r_{j}^{W},r_{j}^{U}|\;I_{j},\theta \right)\\ \;P\left(\mathrm{Ob}s_{j}^{W},\mathrm{Ob}s_{j}^{U}|r_{j}^{W},r_{j}^{U},\theta \right) \end{array}$$

**FIGURE 1:**
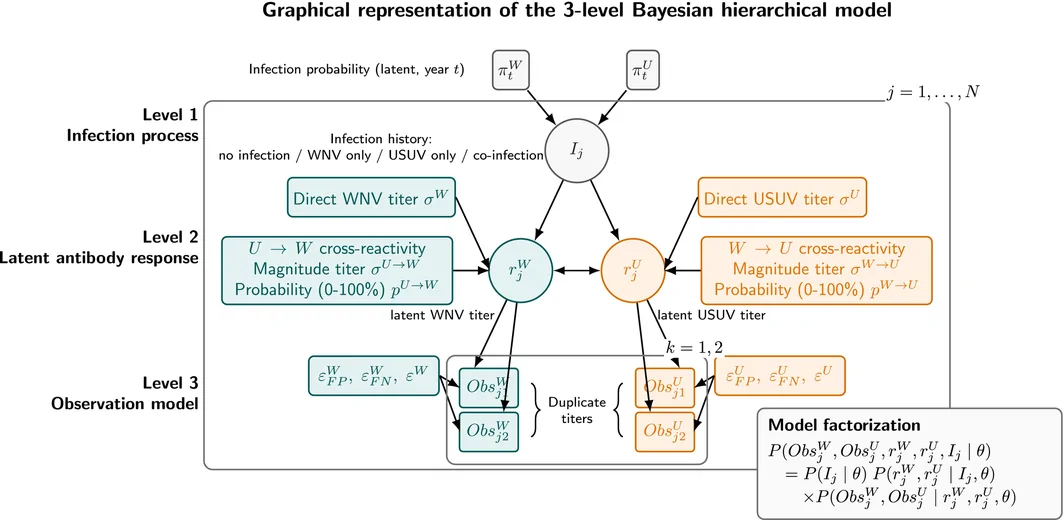
Graphical representation of the 3‐level Bayesian hierarchical model.

In this model, the subscript *j* is used to index the individuals and superscript to indicate the virus (W or U). θ is the vector of parameters. The first term in the hierarchical model, $P\left(I_{j}\;|\theta \right)$, is the probability that among the ELISA positive, an individual had a specific history of infection by WNV and USUV. $I_{j}$ is the infection history which can take four values (0.0), (1.0), (0.1), (1.1), in which the first and second terms characterize whether individual *j* was infected by WNV and USUV, respectively. The second term is the antibody response model that characterizes the distribution of the antibody response for both viruses provided that the infection history is known. We assume antibody responses are due to infection and not maternal antibodies and that neutralizing titers arising from the infection with a virus (and cross‐reactivity) follow a zero‐truncated Poisson distribution. The third term, the observation model, characterizes for each virus how the two observed titers (Obs_*j*_) are distributed with respect to the real (unobserved latent) titre. This term is dependent on the six error terms that describe the probability of false positives ($\varepsilon_{\mathrm{FP}}$), false negatives ($\varepsilon_{\mathrm{FN}}$) and other error (*ɛ*) for WNV and USUV. A more detailed description of the model and parameter estimation can be found in the Supporting Information.

#### 2.3.2 The Likelihood

The model is obtained by summing over all the possible real titre values and all infection histories for individual *j* which can be summarized as:

$$P_{j}=\sum_{I_{j}}\sum_{r_{j}^{W}}\sum_{r_{j}^{U}}P\left(I_{j}\;|\theta \right)\;P\left(r_{j}^{W},r_{j}^{U}|\;I_{j},\theta \right)\;P\left(\mathrm{Ob}s_{j}^{W},\mathrm{Ob}s_{j}^{U}|r_{j}^{W},r_{j}^{U},\theta \right)$$

The loglikelihood is obtained over all individuals and is given by:

$$\mathrm{LL}=\sum_{j}\log P_{j}$$

In addition, negative ELISA assays contribute to the estimation of the error terms ($\varepsilon_{\mathrm{FP}}^{U}$ and $\varepsilon_{\mathrm{FP}}^{W}$). We write $E_{0}$ for the total number of negative ELISA assays, $E_{0}^{W}$ (respectively $E_{0}^{U}$) for the total number of negative ELISA boars that are positive for WNV in VNT (respectively, USUV) and we assume a Binomial distribution for the number of negative ELISA results. We assume a perfect sensitivity of the ELISA (Beck et al. 2017) which means that ELISA negative samples with a positive VNT result are considered false positives. ELISA assays' contribution to the loglikelihood is:

$$\mathrm{LL}=\log P\left(E_{0}^{W};E_{0},\varepsilon_{\mathrm{FP}}^{W}\right)+\log P\left(E_{0}^{U};E_{0},\varepsilon_{\mathrm{FP}}^{U}\right)$$

$$\begin{array}{c} =E_{0}^{W}\log\varepsilon_{\mathrm{FP}}^{W}+\left(E_{0}-E_{0}^{W}\right)\log\left(1-\varepsilon_{\mathrm{FP}}^{W}\right)+E_{0}^{U}\log\varepsilon_{\mathrm{FP}}^{U}\\ \;+\left(E_{0}-E_{0}^{U}\right)\log\left(1-\varepsilon_{\mathrm{FP}}^{U}\right) \end{array}$$

#### 2.3.3 Estimation of Infection Probability From ELISA Assays

For each time period (*t*), we estimate the infection probability from ELISA assays for WNV and USUV $\left(\pi_{t}^{W},\pi_{t}^{U}\right)$, from the estimated posterior probability of positive VNTs $\left(P_{t}^{I}\right)$ among the ELISA positive samples. The ELISA positive tests arise from a binomial process, such that

$${\mathrm{Pos}}_{t}\sim \text{Binomial}\left(E_{\text{total},t},\frac{E_{\mathrm{pos}}{,}_{t}}{E_{\text{total}}{,}_{t}}\right),$$

where $E_{\text{total}}{,}_{t}$ is the total ELISA tests observed and $E_{\mathrm{pos},t}$ is the number of positive ELISA tests observed for either WNV or USUV in period *t*. The overall probability of infection is then given by:

$$\pi_{t}^{I}=P_{t}^{I}\cdot \frac{{\mathrm{Pos}}_{t}}{E_{\text{total},t}\;}.$$

Additionally, we compare the probability of infection as determined by the model $\left(\pi_{t}^{W},\pi_{t}^{U}\right)$ with the classical approach where a four‐fold difference in titre yields a conclusive diagnosis for either WNV or USUV, and a positive result for both agents without sufficient difference an equivocal result.

#### 2.3.4 Parameter Estimation

The priors were set as non‐informative for all parameters used in the model (Supporting Information, Table S2). We fitted the model parameters using a Markov chain Monte‐Carlo (MCMC) framework implemented in the Rstan package. More details on the implementation and diagnostics are shown in the Supporting Information.

#### 2.3.5 Prediction Calculation

We use the model to predict the observed joint response in the case of an infection. When the infection status is known, the probability of observing a set of titers is obtained by accounting for the possible real titers that generated the observation:

$$P\left(\mathrm{Ob}s_{j}^{W},\mathrm{Ob}s_{j}^{U}|I_{j},\theta \right)=\sum_{r_{j}^{W},r_{j}^{U}}P\left(\mathrm{Ob}s_{j}^{W},\mathrm{Ob}s_{j}^{U}\;|r_{j}^{W},r_{j}^{U},I_{j},\theta \right)\;P\left(r_{j}^{W},r_{j}^{U}|I_{j},\theta \right)$$

#### 2.3.6 Sensitivity Analysis

To challenge the assumption of the set cut‐offs and cross‐reactivity, we set scenarios to test the sensitivity of the model results. Changes in data (cut‐off) and model (cross‐reactivity) were combined into three scenarios. The reason for using the different WNV cut‐offs is that an initial cut‐off of 10 was used for WNV, but information from ELISA negative Dutch boars and the set of Norwegian boars informed us about the more appropriate cut‐off of 30 (Figure S2). First, we decreased the VNT cut‐off compared to the baseline model for WNV to 10 (same as USUV), which is called Scenario 1. Furthermore, we tested a scenario in which $p^{W\to U}$ and $p^{U\to W}$ are set to zero, using the cut‐off titre of 30 for WNV and 10 for USUV (Scenario 2). Later, we tested a scenario in which $p^{W\to U}$ and $p^{U\to W}$ are set to zero but using the cut‐off 10 for both WNV and USUV (Scenario 3). All simulations followed the same settings explained in the Supporting Information. Model comparison was performed using the PSIS‐LOO criterion which relies on computing a Bayesian leave‐one‐out estimate of the log pointwise predictive density (LPPD), and to which a standard error is associated. The loo_compare function in the loo package compares the expected log pointwise predictive density (ELPD) and its associated standard error (SE) for the different models and was used for model selection (Vehtari et al. 2017, 2024).

#### 2.3.7 Model Adequacy

Firstly, we assessed the model adequacy by simulating 1000 surveys in a population with the same characteristics as the data (sample size and period). These simulations were compared to the data based on (1) the frequency with which titers were observed and (2) the frequency of concordant samples. After, we used the median estimates of the model's output (baseline and the sensitivity analysis) to generate three datasets. Then, we fit the model using the in silico data and assessed whether the outputs contain the parameters within the 95% credible intervals.

## 3 Results

A total of 1504 wild boar sera, collected between January 1st 2018 and December 31st 2021, were randomly selected from the sample database. A total of 372, 390, 354 and 388 samples were selected from 2018 to 2021, respectively. These comprised 41.69% females, 44.74% males and 13.56% boars with unreported sex. The location, sex and age group of sampled wild boars is shown in Figure 2. A more detailed description of the sample characteristics is available in Table S3 (Supporting Information).

**FIGURE 2:**
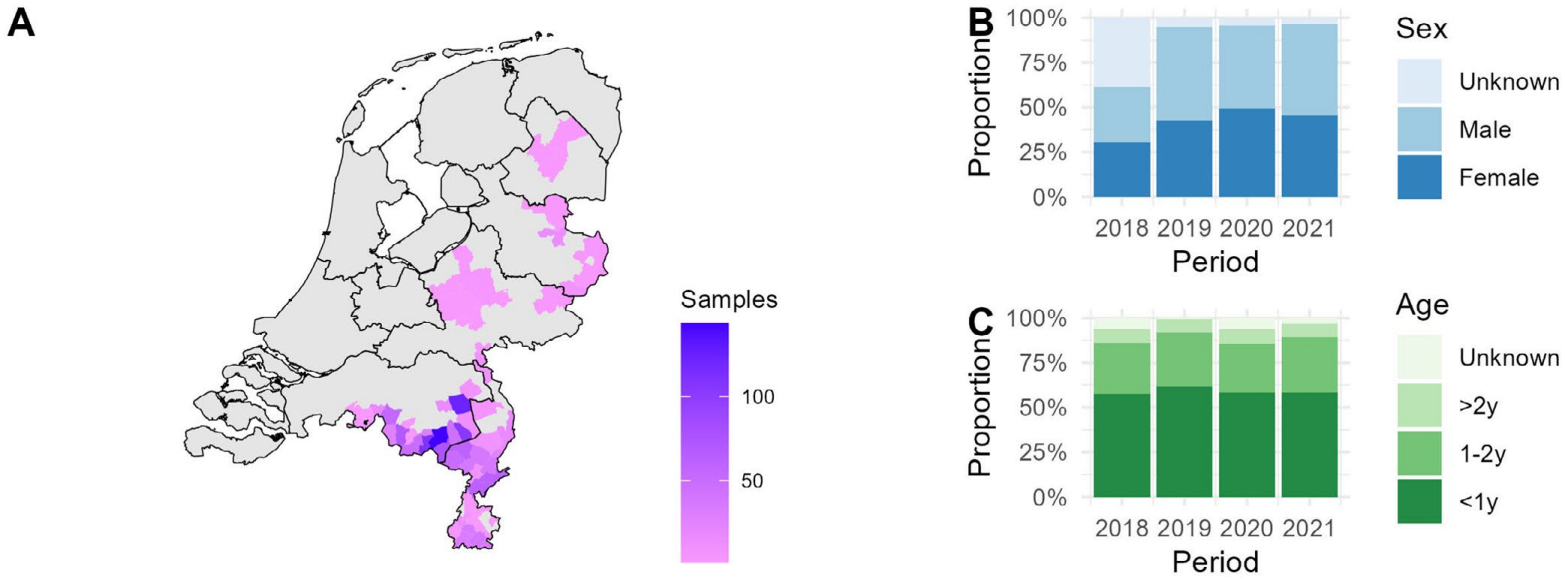
(A) Location of sampled wild boars over the study period, (B) Sex of wild boars per year, (C) Age groups of wild boars per year. Total number of animals per year: 372 (2018), 390 (2019), 354 (2020) and 388 (2021).

### 3.1 Serology

In total, 30.65% (461/1504) of sera had a doubtful or positive ELISA result. A declining trend in ELISA positives was observed over time; from 44.89% (167/372) to 31.54% (123/390), 25.71% (91/354) and 20.62% (80/388) per year for 2018 to 2021, respectively. Five ELISA positive samples, three from 2018, one from 2019 and one from 2021, could not be tested in VNTs due to insufficient remaining volume. VNTs could not be interpreted (titre < 90 or < 270) in 5, 7, 31, and 8 samples from 2018, 2019, 2020 and 2021, respectively. These 55 ELISA positive sera (12.15%, 56/461) had to be excluded from further analyses. Thus, 405 ELISA positive individuals were included in the statistical model. Of these, 159, 115, 60, and 71 were from 2018 to 2021, respectively.

The VNT titre distribution of these 405 ELISA positive individuals is shown in Figure 3. Based on the classical interpretation (four‐fold titre difference), a declining trend in USUV positives was observed over the years (Figure 4A, Table S4). The proportions of equivocal (less than four‐fold titre difference) individuals were 21.24%, 11.03%, 5.08% and 8.25% from 2018 to 2021, respectively.

**FIGURE 3:**
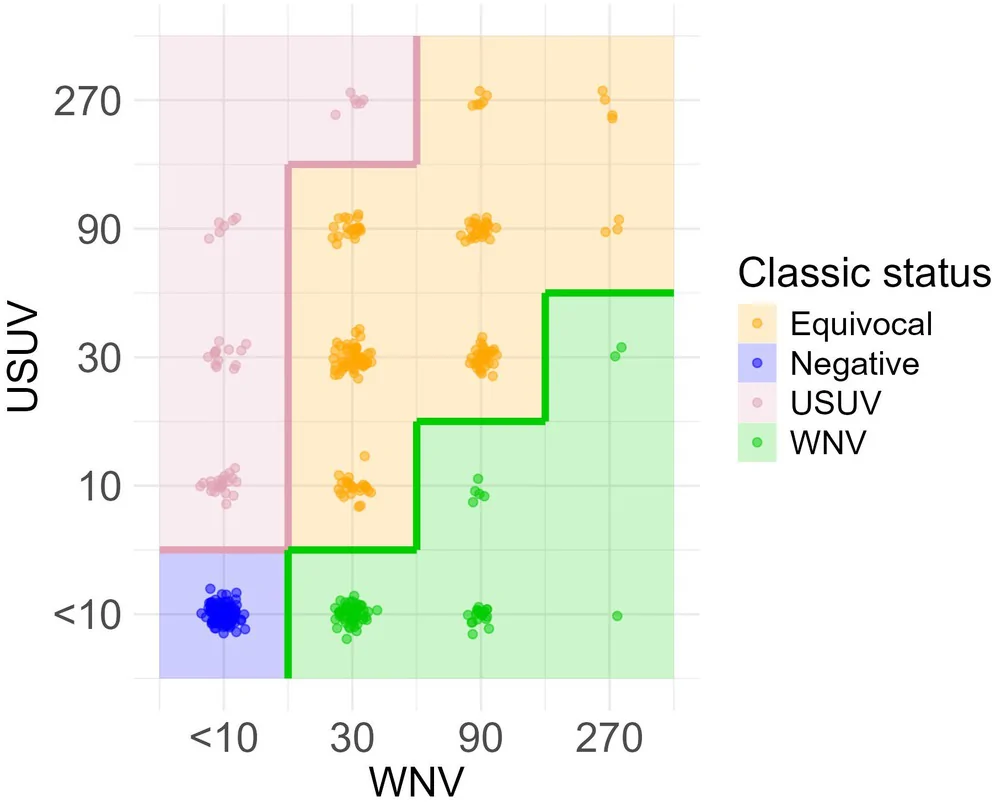
VNT titre distribution and classification by the classical approach (four‐fold titre difference) based on the mean titre of two duplicates for all animals included in the model (*n* = 405).

**FIGURE 4:**
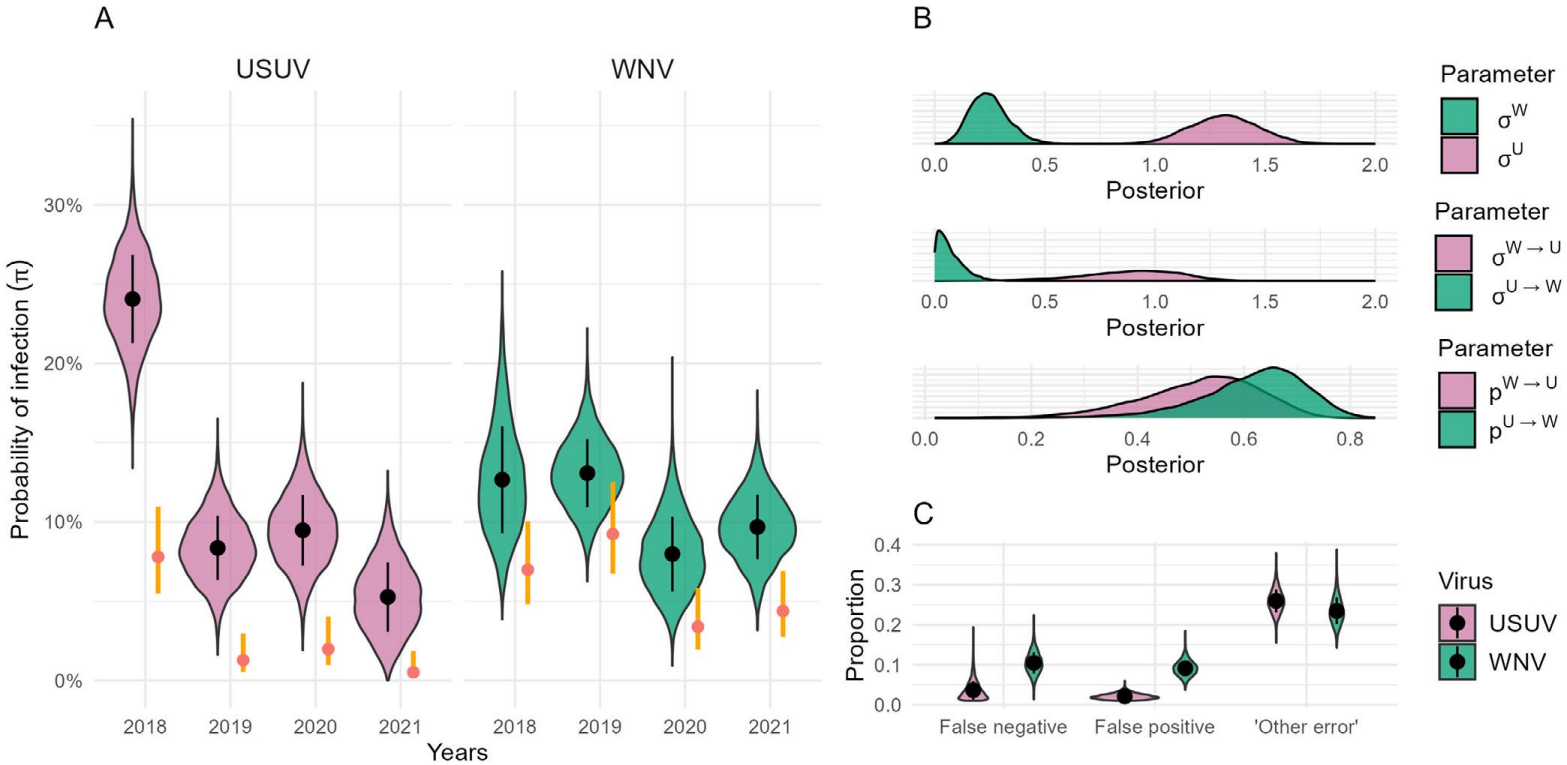
(A) Probability of infection (*π*) in or before year (t) of WNV and USUV in wild boars from 2018 to 2021 in the Netherlands. The orange ranges are the observed prevalence and 95% confidence interval (calculated according to Wilson's method) based on the classical interpretation (four‐fold titre difference). (B) Direct and indirect titre response (top and middle) and the probability of cross‐reactivity (lower). (C) Observational errors: Probabilities of false positives ($\varepsilon_{\mathrm{FP}}$), false negatives ($\varepsilon_{\mathrm{FN}}$) and other errors (*ɛ*) for USUV and WNV.

### 3.2 Model Results

We found that the baseline model taking into account cross‐reactivity outperformed the scenarios without cross‐reactivity. Results of the sensitivity analyses and LOOIC comparison of scenarios are included in the Supporting Information (Figure S3, Table S5).

With regards to the probabilities of infection prior to sampling, we observe similar trends compared to using the classical interpretation approach. However, the actual estimated probabilities are higher for both viruses and all years in the model compared to the classical interpretation, especially for USUV in 2018 (Figure 4A).

The expected titre following infection with WNV was 39.97 (33.49–48.42, 95% CrI), and 42.41 (30.83–58.45, 95% CrI) for USUV. Infection with WNV increased USUV titers by 27.21 (15.23–40.01, 95% CrI), while infection with USUV increased WNV titers by 31.87 (30.07–37.79, 95% CrI). These titre values were obtained by transforming the posterior densities observed in Figure 4B according to 10.3^σ^, for USUV and 10.3^(*σ*+1)^ for WNV. The probability of a cross‐reactive (USUV) response after WNV infection was 52.99% (26.57%–69.69%), while the probability of a cross‐reactive (WNV) response after USUV infection was higher, namely 63.17% (38.38%–77.23%) (Figure 4B).

The probability of false positives ($\varepsilon_{\mathrm{FP}}$) was higher for WNV (9.07%, 5.71%–13.03%, 95% CrI) than for USUV (2.00%, 1.08%–3.76%, 95% CrI) (Figure 4C). The probability of false negatives ($\varepsilon_{\mathrm{FN}}$) for WNV was 10.35% (5.63%–15.88%, 95% CrI) and for USUV 3.00% (1.08%–9.54%, 95% CrI). The probability of other observational errors (ε) for WNV was 23.23% (17.66%–30.54%, 95% CrI) and 25.86% (20.87%–31.97%, 95% CrI) for USUV (Figure 4C).

Next, we used the model results to simulate antibody responses following infection and to assess the performance of the classical method. We accounted for the prediction that USUV infection will lead to an increase in WNV titre in 63% of cases, and WNV infection will result in USUV titre in 53% of cases (Figure 3B). These direct and cross‐reactive responses will lead to an equivocal response in a large proportion of single‐virus infections (Figure 5, Table S4). In co‐infected animals, 68% would be classified as equivocal based on the classical interpretation (Figure 5B).

**FIGURE 5:**
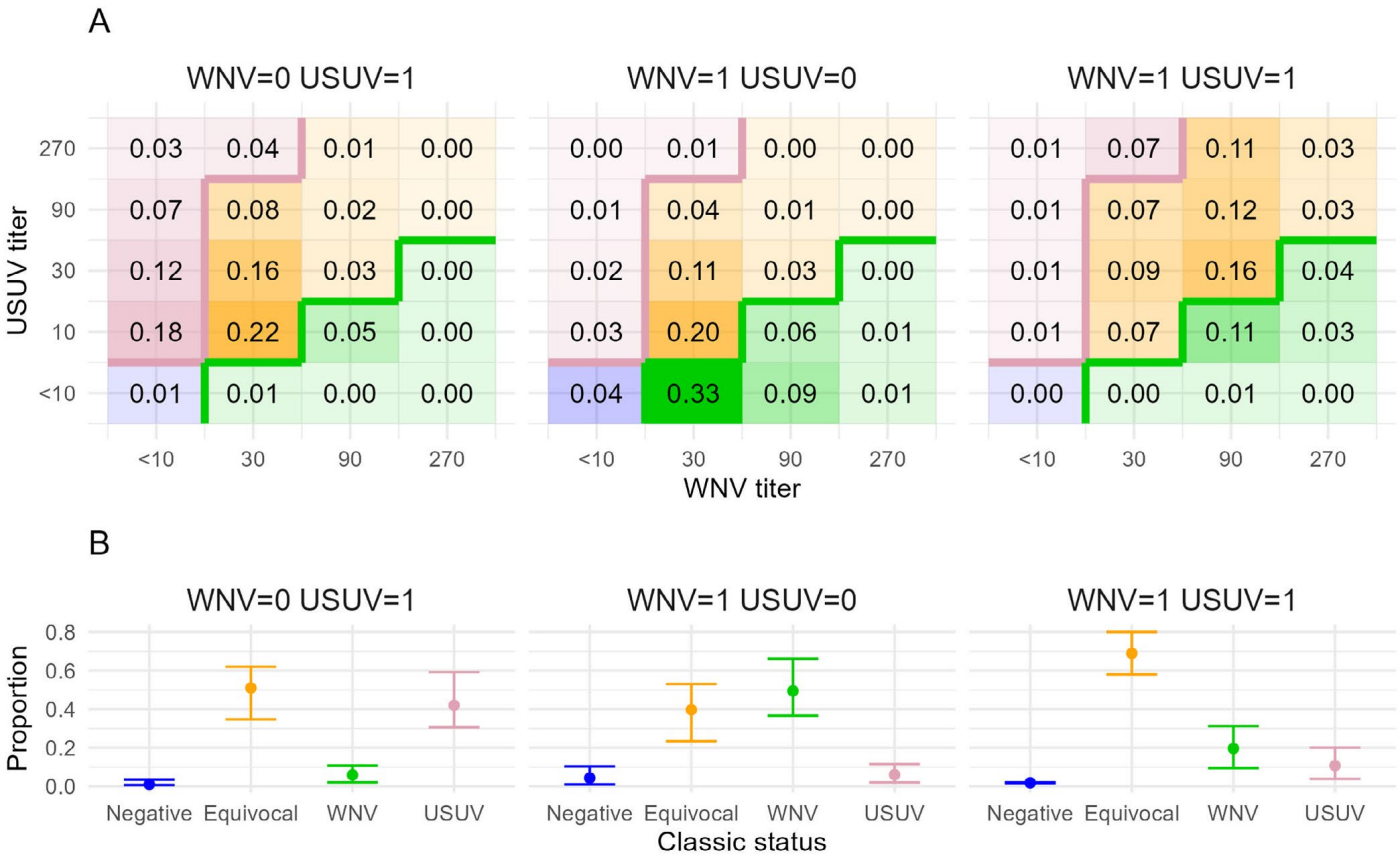
(A) Proportion of animals with respective real titers $r_{j}^{W}$ and $r_{j}^{U}$ (WNV on *x*‐axis, USUV on *y*‐axis) after single (left and centered panel) or co‐infection (right panel). USUV or WNV = 1, means positive status for infection and USUV or WNV = 0, means negative status for infection. Colour code is given in the legend of Figure 2. (B) Proportion of animals classified as negative, equivocal (less than four‐fold titre difference), WNV or USUV infected based on their real titers (panel A).

The posterior distributions of all parameters and model diagnostics are provided in the–(Figures S4–S8, Table S6).

## 4 Discussion

With this study we provide epidemiological insights into USUV and WNV infections in wild boars in the Netherlands over the years 2018–2021. We found previously undetected infections of WNV in wild boars in, or before, 2018. We found that cross‐reactivity impaired the interpretation of the serological results, but the model provided insight in the development of direct and cross‐reactive responses in addition to observational errors for USUV and WNV in wild boars. Despite the seemingly similar conclusions drawn from the classical serological interpretation versus our model results with regards to the infection status, the use of the Bayesian model offers much more insight in infection patterns for animals that show neutralizing titers for both viruses. The increased understanding of the direct and cross‐reactive titre development and probabilities of observational errors exposes and quantifies uncertainties the classical approach cannot capture.

The high proportion of wild boars with neutralizing antibody titers throughout our study period was surprising. This is in contrast to findings in other European countries such as Poland, where no ELISA positives were detected in 2017–2018 (Niczyporuk and Jabłoński 2021). A serosurvey in equines and dogs in the Netherlands in 2021–2022 also revealed considerably lower percentages of seropositive animals (Streng, Hakze‐van der Honing, et al. 2024). Furthermore, we show evidence of possible WNV exposure in animals before the first official detection of the virus in 2020 (Sikkema et al. 2020). Both the classical interpretation as well as the model results indicate a WNV probability of infection of at least 3%, thus providing evidence of WNV introduction(s) in or before 2018. In 2018, a large outbreak of WNV caused over 1500 human cases in the European Union and the virus was first found in Germany (ECDC 2019). The warm summer of 2018 provided the optimal conditions for WNV circulation, which could also explain potential introduction(s) into the Netherlands (De Freitas Costa et al. 2024). West Nile virus seropositivity has been detected before 2017 in (migrating) wild birds, but virus detection was not reported until 2020 (Münger et al. 2025; Sikkema et al. 2020). Our findings could indicate that WNV was introduced locally, but potentially no domestic animals or humans got infected. Additionally, even though wild boar have a limited home range, we cannot be certain boars in our study were indeed infected in the Netherlands as their home range may include parts of Germany, Belgium or Luxemburg (Fattebert et al. 2017; Jánoska et al. 2018; Miettinen et al. 2023). For USUV we observe a declining trend with regards to the probability of infections, which could be due to the large outbreak in 2016 followed by lower circulation in the years after as was also observed in wild bird populations (Münger et al. 2025; Rijks et al. 2016).

Wild boars have been part of surveillance systems for a wide range of (zoonotic) pathogens in many countries for decades (Abrantes and Vieira‐Pinto 2023; Zanet et al. 2024). Their potentially high exposure to vectors, limited home range, as well as the fact that they are not vaccinated makes wild boars suitable for surveillance of orthoflaviviruses. Even though pathogen detection in wild boars is usually not achieved, the dead‐end host may still be a valuable sentinel to detect past infections (Escribano‐Romero et al. 2015; Grassi et al. 2023; Halliday et al. 2007; Racloz et al. 2006). In the Netherlands, wild boar samples are collected each year for other surveillance purposes (Streng et al. 2023). Disadvantages of the use of wild boars are poor serum sample quality and the limited geographical range of wild boars in the Netherlands. Nevertheless, even in 2018 when there were no samples from the southern‐most province (Limburg) we found evidence for WNV infections. Surveillance of wild boars could be supplemented by increasing sampling efforts of other (dead‐end) hosts such as wild birds, equines or chickens around the seropositive wild boars to further investigate local circulation of USUV and WNV.

This study has several limitations that should be considered when interpreting the findings. Firstly, even though our model provides new insights, no modelling approach can completely resolve the issue of cross‐reactivity and its exact effects on the estimated probability of (past) infection as described here. The results were further complicated by the absence of negative and positive control sera, which are vital to better interpret serological results in the future. Ideally, samples of confirmed USUV infected but WNV negative wild boars, and vice versa, would be available to further study probabilities and magnitude of cross‐reactions in both directions. This confirmation could be either based on circulation of only one of two viruses in a specific area, a positive PCR test or repeated serological testing after infection. This highlights another limitation, as RNA detection is usually not achieved in this surveillance system, and therefore timing of infection is uncertain. All probabilities of infection should therefore be interpreted with caution, as they do not necessarily reflect seroprevalence.

## Author Contributions

Conceptualization: Kiki Streng. Methodology: Kiki Streng, Eduardo de Freitas Costa, Nathanaël Hozé, Michel Counotte. Software: Eduardo de Freitas Costa, Nathanaël Hozé, Michel Counotte. Formal analysis: Eduardo de Freitas Costa, Nathanaël Hozé, Michel Counotte. Investigation: Kiki Streng, Sophie van Oort, Renate Hakze‐van der Honing. Resources: Gebbiena Bron, Carl Andreas Grøntvedt, Nathanaël Hozé. Data curation: Kiki Streng, Eduardo de Freitas Costa, Nathanaël Hozé, Michel Counotte. Writing – original draft: KS, Eduardo de Freitas Costa, Nathanaël Hozé, Michel Counotte. Writing – review and editing, Visualization: Sophie van Oort, Renate Hakze‐van der Honing, Gebbiena Bron, Carl Andreas Grøntvedt, Wim H.M. van der Poel. Supervision: Wim H.M. van der Poel. Project administration: Kiki Streng. Funding acquisition: WP.

## Funding

The research described in this thesis was financially supported by the project ‘Preparing for vector‐borne virus outbreaks in a changing world: a One Health Approach’ (NWA.1160.1S.210), which is (partly) financed by the Dutch Research Council (NWO). The project also received funding from the Dutch Ministry of Agriculture, Fisheries, Food Security and Nature (LVVN) within the Wageningen University and Research Statutory Tasks, project Preparing for Emerging Pathogens (project number 1600003002).

## Ethics Statement

All animals included were sampled after death, as part of ongoing surveillance or slaughterhouse monitoring. Therefore, no ethical approval was needed for the use of these samples.

## Conflicts of Interest

The authors declare no conflicts of interest.

## Supporting information


**Table S1:** Conditional probability of observing a titre value for all combinations included in the observation model. The probabilities that are not mentioned are equal to 0.
**Table S2:** Overview of model parameters and their prior distribution/calculation and description.
**Figure S1:** ELISA results of the negative control groups (ELISA neg. boar: random selection of wild boars from our dataset that were ELISA negative, NVI = Norwegian wild boars sampled in 2018–2019, commercial pigs = Dutch slaughterhouse samples from commercial pigs). S/N% values < 50 are considered positive according to the manufacturer's instructions.
**Figure S2:** VNT results of the negative control groups for Usutu and West Nile virus, dashed lines indicate cut‐offs for both VNTs (ELISA neg. boar: random selection of wild boar from our data that were ELISA negative, NVI = Norwegian wild boars sampled in 2018–2019, commercial pigs = Dutch slaughterhouse samples from commercial pigs).
**Table S3:** Descriptive results of wild boars ( *Sus scrofa* ) included in the study per year. ^*1*^Mean *(IQR)*; ^*2*^ *n (%)*.*In 2018 and 2021, limited/no sera were collected in the province of Limburg, due to shortage in personnel.
**Table S4:** Results of the 1504 wild boar serum samples from 2018 up to 2021 in the Netherlands based on a four‐fold VNT titre difference. Titres of < 10 and < 30 are regarded negative for both USUV and WNV. Cut‐off for the VNT is a titre of 10 for USUV and 30 for WNV. Bad serum quality includes all ELISA positive boars with VNT values of < 90 or < 270 or not able to be tested in VNT at all.
**Figure S3:** Posterior distributions for titers $\sigma^{W}$, $\sigma^{U}$ (A) and $\lambda^{I}$ (infection rate) over 2018, 2019, 2020, and 2021 (B). The values are the posterior for: Scenario 1 (WNV and USUV cut‐off at 10), Scenario 2 (no cross‐reaction, WNV and USUV cut‐off at 10), and Scenario 3 (no cross‐reaction, cut‐offs same as baseline). The observation errors $\varepsilon^{W}$, $\varepsilon^{U}$, $\varepsilon_{\mathrm{FN}}^{W}$, $\varepsilon_{\mathrm{FN}}^{U}$ and $\varepsilon_{\mathrm{FP}}^{W}$, $\varepsilon_{\mathrm{FP}}^{U}$ showed minor changes compared to the baseline and were omitted.
**Figure S4:** Comparison of the model predicted titre responses (model) and the observed titre response (data) for Usutu virus (USUV) and West Nile virus (WNV).
**Figure S5:** Proportion concordant and 95% credible interval of replicates for USUV (A) and WNV (B). The x‐axis shows the lowest titre value of the replicate.
**Figure S6:** Posterior distribution (median and 95% credible interval) of the model parameters obtained by fitting the baseline model to the in silico data. The grey dots are the in silico parameters used to generate the dataset and the pink interval is the 95% credible interval obtained by fitting the data.
**Table S5:** Model fitness comparison using LOOIC in four different models used in the sensitivity analysis.
**Figure S7:** Comparison of prior and posterior distribution for model parameters.
**Table S6:** Posterior summary statistics for all model parameters estimated via sampling (4 chains × 10,000 post‐warmup iterations). mean: posterior mean of the parameter across all retained samples; serves as the point estimate; sd: posterior standard deviation; quantifies uncertainty around the mean estimate; n_eff: effective sample size; Rhat: Gelman–Rubin potential scale reduction factor; compares within‐chain to between‐chain variance. Values at or near 1.00 indicate that all chains have converged to the same distribution.

## Data Availability

The datasets used and analysed during the current study are available from the corresponding author on reasonable request.
