# Supplementary material for: Evidence of West Nile Virus Infections in Wild Boars (Sus scrofa) in the Netherlands, 2018–2021
Source: Zoonoses Public Health. 2026 Sep 6;73(7):670–9. doi: 10.1111/zph.70088 (PMC13633445; doi:10.1111/zph.70088)
Supplement: Supplementary file 1 [file ZPH-73-670-s001.docx]

# Supplementary material

**Methods**

*Notations*

All animals were individually identified by a unique number. All animals are assumed to be independent of each other. Demographic information and test results were stored in Excel (version 2308). Virus Neutralization Tests (VNT) results (neutralizing antibody titers) can take the values (10, 30, 90 or ≥270) which we write with an exponent form 10*3^o^ for USUV and 10*3^o+1^ for WNV. All titers are thus given a label which can be: *o* = 0, 1, 2, 3, 4 for USUV and *o* = 0, 1, 2, 3 for WNV. VNT titers of <10 and <30 (where the 1:10 dilution was not interpretable) are set to 0 (negative) for both USUV and WNV. As all individuals were tested in duplicate in both VNTs, we write $Obs^{W}=\left( o_{1}^{W}, o_{2}^{W} \right)$ and $Obs^{U}= \left( o_{1}^{U}, o_{2}^{U} \right)$ for the set of observed WNV and USUV titers, respectively.

*The observation model*

The observation model characterizes how the observed titers are distributed with respect to the real (unobserved latent) titer. The ‘real’ titers for individual *j* for WNV and USUV are noted $r_{j}^{W}$ and $r_{j}^{U}$, respectively. We make several assumptions. First, observed values are at most one titer unit apart from the real value. Therefore, if the real titer is *r*, the observed value can be *r*-1, *r*, or *r*+1. We distinguish the parameters characterizing the occurrence of false negatives${(\varepsilon}_{fn})$ and false positives ($\varepsilon_{fp})$ from other types of errors ($\frac{\varepsilon}{2}$). Two replicates of the observed values are available for each individual which may differ from the real titers. We consider that the observations are independent so that:

$$P\left( Obs_{j}^{W}, Obs_{j}^{U} | r_{j}^{W}, r_{j}^{U}, \theta\right) =P\left( o_{1}^{W} | r_{j}^{W}, \theta\right) .P\left( o_{2}^{W} | r_{j}^{W}, \theta\right).P\left( o_{1}^{U} | r_{j}^{U}, \theta\right).P\left( o_{2}^{W} | r_{j}^{U}, \theta\right)$$

These terms depend on the six error terms $\varepsilon^{W}, \varepsilon_{FP}^{W}, \varepsilon_{FN}^{W}, \varepsilon^{U}, \varepsilon_{FP}^{U}, \varepsilon_{FN}^{U}$ and are detailed in Table S1. By assuming that the observations are at most one titer value away from the real value, the replicates can be equal, different by one titer, or different by two titer values. For instance, if the real value is 2 (*r* = 2), the observed pair of titers can be either (1,1), (1,2), (2,1), (2,2), (3,2), (2,3), (3,1) or (1,3). Conversely, if for instance the observed titers are (1,2), the possible real value is 1 or 2, but not 3 because 1 and 3 are two titers apart. Moreover, we account for right-censoring for real titers larger than 4. In the conditional observation probability, we write $P\left( 4 | r,\theta\right)=1$.

**Table S1**. Conditional probability of observing a titer value for all combinations included in the observation model. The probabilities that are not mentioned are equal to 0.

| **Observed (**$\boldsymbol{o}$**)** | **Real (**$\boldsymbol{r}$**)** | **Probability (**$\boldsymbol{P(o\vert r)}$**)** |
| --- | --- | --- |
| 0 | 0 | $1-\varepsilon_{FP}$ |
| 1 | 0 | $\varepsilon_{FP}$ |
| 0 | 1 | $\varepsilon_{FN}$ |
| 1 | 1 | $1-\varepsilon_{FN}-\frac{\varepsilon}{2}$ |
| 2 | 1 | $\frac{\varepsilon}{2}$ |
| 1 | 2 | $\frac{\varepsilon}{2}$ |
| 2 | 2 | $1-\varepsilon$ |
| 3 | 2 | $\frac{\varepsilon}{2}$ |
| 2 | 3 | $\frac{\varepsilon}{2}$ |
| 3 | 3 | $1-\varepsilon$ |
| 4 | 3 | $\frac{\varepsilon}{2}$ |
| 3 | 4 | $\frac{\varepsilon}{2}$ |
| 4 | 4 | $1-\frac{\varepsilon}{2}$ |
| 4 | >4 | 1 |

*The infection model*

The first term in the hierarchical model, $P\left( I_{j} | \theta\right)$, is the probability that among the ELISA positive, an individual had a specific history of infection by WNV and USUV. Assuming that the probability of infection by both viruses are independent, at each period, the relation between the probability of infection and infection status is given by the set of four equations:

Not previously infected:	$P_{t}\left( I = \left( 0,0 \right) | \theta\right) = \left( 1-P_{t}^{W} \right). \left( 1-P_{t}^{U} \right)$

Previously WNV infected:	$P_{t}\left( I = \left( 1,0 \right) | \theta\right) = P_{t}^{W}. \left( 1-P_{t}^{U} \right)$

Previously USUV infected:	$P_{t}\left( I = \left( 0,1 \right) | \theta\right) = \left( 1-P_{t}^{W} \right) .P_{t}^{U}$

Previously WNV and USUV infected:	$P_{t}\left( I = \left( 1,1 \right) | \theta\right) = P_{t}^{W}.P_{t}^{U}$

*The antibody response model*

The antibody response model characterizes the distribution of the antibody response for both viruses provided that the infection history is known. We assume antibody responses are due to infection and not maternal antibodies and that neutralizing titers arising from the infection with a virus (and cross-reactivity) follow a zero-truncated Poisson distribution.

The model extends the one developed in Hozé et al. (16) and we recall the general line here. If individual *j* has not been infected by either WNV or USUV, they have no response and the probability of having titers $r_{j}^{W}$ and $r_{j}^{U}$ is $P\left( r_{j}^{W}, r_{j}^{U} | I_{j} = \left( 0,0 \right), \theta\right)=1$ and in case $r_{j}^{W}$ and/or $r_{j}^{U}$≠ 0, $P\left( r_{j}^{W}, r_{j}^{U} | I_{j} = \left( 0,0 \right), \theta\right)=0$. In case of the other infection history options, i.e., $I_{j} = \left( 1,0 \right)$, $I_{j} = \left( 0,1 \right)$ and $I_{j} = \left( 1,1 \right)$, the model follows as described in Hozé et al. (16) and includes the direct and cross-reactive response due to infection given by a zero-truncated Poisson distribution. In the case of an infection by WNV and not by USUV, the direct response is indicated by the parameter $\sigma^{W}$. A cross-reactive USUV response occurs with a probability $p^{W\to U}$, and the titer increase is given by the parameter $\sigma^{W\to U}.$Moreover, we assume that the cross-reactive response does not exceed the direct response and introduce a parameter $f^{W\to U}\leq1$ such that $\sigma^{W\to U}=f^{W\to U}\times\sigma^{W}.$ In the case of an infection with both viruses, both the direct and the cross-reactive responses contribute to the titer increase.

*Parameter estimation*

A No U-Turn sampler variant of Hamiltonian Monte-Carlo was used to update the parameters. Two chains of 15000 iterations with a burnin of 5000 iterations were ran, thinning each 3 samples. The analyses were run in R Core Team (2023). Model diagnostics were assessed with the trace plot, Gelman Rubin plots, and potential scale reduction factors convergence diagnostic. The diagnostics show that all models converged properly. A full overview of model parameters is shown in Table S2.

**Table S2.** Overview of model parameters and their prior distribution/calculation and description

| **Category** | **Parameter** | **Prior distribution**  **/ calculation** | **Description** |
| --- | --- | --- | --- |
| Cross-Reactivity | $p^{W\to U}$ | $\sim U(0, 1)$ | Probability WNV infection causes cross-reactivity in USUV assay |
|  | $p^{U\to W}$ | $\sim U(0, 1)$ | Probability USUV infection causes cross-reactivity in WNV assay |
|  | ${prop}^{W\to U}$ | $\sim U(0, 1)$ | Proportion scaling $\sigma^{W\to U}$from $\sigma^{U}$ |
|  | ${prop}^{U\to W}$ | $\sim U(0, 1)$ | Proportion scaling $\sigma^{U\to W}$from $\sigma^{W}$ |
| Titer Response | $\sigma^{W}$ | $\sim U(0, 4)$ | Mean WNV titer response |
|  | $\sigma^{U}$ | $\sim U(0, 4)$ | Mean USUV titer response |
|  | $\sigma^{W\to U}$ | ${prop}^{W\to U}\times\sigma^{W}$ | **Transformed:** Cross-reactive WNV to USUV titer scale |
|  | $\sigma^{U\to W}$ | ${prop}^{U\to W}\times\sigma^{U}$ | **Transformed:** Cross-reactive USUV to WNV titer scale |
| Probability of Infection | $\lambda_{t}^{W}$ | $\sim U(0, 10)$ | Infection rate (per period, t=1-4) for WNV |
|  | $\lambda_{t}^{U}$ | $\sim U(0, 10)$ | Infection rate (per period, t=1-4) for USUV |
|  | $P_{t}^{W}$ | $1-e^{-\lambda_{t}^{W}}$ | Prevalence term (per period, t=1-4) for WNV |
|  | $P_{t}^{U}$ | $1-e^{-\lambda_{t}^{U}}$ | Prevalence term (per period, t=1-4) for USUV |
|  | $\pi_{t}^{W}$ | $P_{t}^{W}\cdot\frac{{Pos}_{t}}{E_{total, t}}$ | Overall probability of infection (per period, t=1-4) for WNV |
|  | $\pi_{t}^{U}$ | $P_{t}^{U}\cdot\frac{{Pos}_{t}}{E_{total, t}}$ | Overall probability of infection (per period, t=1-4) for USUV |
| Observation Error | $\varepsilon^{W}$ | $\sim U(0.01, 0.99)$ | Conditional probability of a ±1 measurement error for WNV |
|  | $\varepsilon^{U}$ | $\sim U(0.01, 0.99)$ | Conditional probability of a ±1 measurement error for USUV |
|  | $\varepsilon_{FP}^{W}$ | $\sim U(0.01, 0.99)$ | False positive rate, WNV |
|  | $\varepsilon_{FP}^{U}$ | $\sim U(0.01, 0.99)$ | False positive rate, USUV |
|  | $f^{W}$ | $\sim U(0.01, 0.99)$ | Scaling factor for WNV false negative rate |
|  | $f^{U}$ | $\sim U(0.01, 0.99)$ | Scaling factor for USUV false negative rate |
|  | $\varepsilon_{FN}^{W}$ | $f^{W}\times\varepsilon^{W}$ | **Transformed:** WNV False negative rate |
|  | $\varepsilon_{FN}^{U}$ | $f^{W}\times\varepsilon^{U}$ | **Transformed:** USUV False negative rate |
| Virus Prevalence | ${prev\_virus}_{t}$ | $\sim U(0.01, 0.99)$ | Underlying virus prevalence for each period (t=1-4), based on ELISA prevalence ($\frac{{E_{pos},}_{t}}{{E_{total},}_{t}}$) |

**Serology**

**Figure S1.** ELISA results of the negative control groups (ELISA neg. boar: random selection of wild boars from our dataset that were ELISA negative, NVI= Norwegian wild boars sampled in 2018-2019, commercial pigs = Dutch slaughterhouse samples from commercial pigs). S/N% values <50 are considered positive according to the manufacturer’s instructions.

**Figure S2.** VNT results of the negative control groups for Usutu and West Nile virus, dashed lines indicate cut-offs for both VNTs (ELISA neg. boar: random selection of wild boar from our data that were ELISA negative, NVI= Norwegian wild boars sampled in 2018-2019, commercial pigs = Dutch slaughterhouse samples from commercial pigs).

**Results**

**Table S3.** Descriptive results of wild boars (*Sus scrofa*) included in the study per year. *^1^ Mean (IQR); ^2^ n (%).*In 2018 and 2021, limited/no sera were collected in the province of Limburg, due to shortage in personnel.*

| **Characteristic** | **Year** | | | |
| --- | --- | --- | --- | --- |
|  | 2018, N = 372 | 2019, N = 390 | 2020, N = 354 | 2021, N = 388 |
| Age in months | 13 (6, 18)^1^ | 13 (5, 18)^1^ | 14 (6, 18)^1^ | 14 (8, 18)^1^ |
| Unknown | 23 | 2 | 30 | 13 |
| Age group | | | | |
| 1-12 months | 214 (61%)^2^ | 241 (62%)^2^ | 201 (62%)^2^ | 226 (60%)^2^ |
| 13-24 months | 107 (31%) | 118 (30%) | 94 (29%) | 120 (32%) |
| 25-36 months | 24 (6.9%) | 22 (5.7%) | 17 (5.2%) | 19 (5.1%) |
| >36 months | 4 (1.1%) | 7 (1.8%) | 12 (3.7%) | 10 (2.7%) |
| Unknown | 23 | 2 | 30 | 13 |
| Sex | | | | |
| Female | 113 (50%) | 167 (45%) | 170 (52%) | 177 (47%) |
| Male | 113 (50%) | 203 (55%) | 160 (48%) | 197 (53%) |
| Unknown | 146 | 20 | 24 | 14 |
| Province* | | | | |
| Drenthe | 0 (0%) | 1 (0.3%) | 1 (0.3%) | 0 (0%) |
| Gelderland | 4 (2.8%) | 3 (0.8%) | 16 (4.6%) | 0 (0%) |
| Limburg | 2 (1.4%) | 211 (55%) | 114 (33%) | 0 (0%) |
| Noord-Brabant | 137 (94%) | 156 (41%) | 199 (58%) | 387 (100%) |
| Overijssel | 2 (1.4%) | 11 (2.9%) | 15 (4.3%) | 0 (0%) |
| Unknown | 227 | 8 | 9 | 1 |

**Table S4.** Results of the 1504 wild boar serum samples from 2018 up to 2021 in the Netherlands based on a four-fold VNT titre difference. Titres of <10 and <30 are regarded negative for both USUV and WNV. Cut-off for the VNT is a titer of 10 for USUV and 30 for WNV. Bad serum quality includes all ELISA positive boars with VNT values of <90 or <270 or not able to be tested in VNT at all.

| Results | 2018 | | 2019 | | 2020 | | 2021 | |
| --- | --- | --- | --- | --- | --- | --- | --- | --- |
|  | **N** | **%** | **N** | **%** | **N** | **%** | **N** | **%** |
| USUV | 40 | 10,75 | 13 | 3,33 | 9 | 2,54 | 6 | 1,55 |
| WNV | 13 | 3,49 | 20 | 5,13 | 6 | 1,69 | 13 | 3,35 |
| Equivocal | 60 | 16,13 | 33 | 8,46 | 16 | 4,52 | 27 | 6,96 |
| Bad serum | 9 | 2,42 | 8 | 2,05 | 31 | 8,76 | 9 | 2,32 |
| Negative | 250 | 67,20 | 316 | 81,03 | 292 | 82,49 | 333 | 85,82 |
| Total | 372 | 100 | 390 | 100 | 354 | 100 | 388 | 100 |

**Sensitivity analysis**

Model 1 (WNV and USUV cut-off at 10) results in higher median cross-reactivity titers from WNV to USUV ($\sigma^{W\to U}$), namely 0.43 instead of 0.05 in the baseline model. The median cross-reactivity titers from USUV to WNV ($\sigma^{U\to W}$) was lower in model 1 compared to baseline (0.32 instead of 0.91). The probability of cross-reaction from WNV to USUV ($p^{W\to U}$) decreased from 0.53 to 0.21, and the probability of cross-reaction from USUV to WNV ($p^{U\to W}$) showed a increse, from 0.63 to 0.81. As model 2 and 3 represent a scenarios without cross-reactivity, cross-reactivity titers and the probability of cross-reaction are not depicted in Figure 4.

For both model 1 and model 3, the VNT cut-off for WNV was reduced from 30 to 10. This results in an increase in the antibody response for WNV, but has limited effect on USUV antibody response besides increased titers for models without cross-reaction (Figure 4A). For the observational errors, all models lead to minor changes compared to the baseline (less than 3%) and the results were omitted from Figure 4. It is observed that the baseline model has lower $\lambda_{t}^{I}$ in all years compared to the other scenarios for both diseases (Figure 4B).

**Figure S3.** Posterior distributions for titers $\sigma^{W}$, $\sigma^{U}$ (A), and $\lambda^{I}$ (infection rate) over 2018, 2019, 2020, and 2021 (B). The values are the posterior for: scenario 1 (WNV and USUV cut-off at 10), scenario 2 (no cross-reaction, WNV and USUV cut-off at 10), and scenario 3 (no cross-reaction, cut-offs same as baseline). The observation errors $\varepsilon^{W}$, $\varepsilon^{U}$, $\varepsilon_{FN}^{W}$, $\varepsilon_{FN}^{U}$, and $\varepsilon_{FP}^{W}$, $\varepsilon_{FP}^{U}$ showed minor changes compared to the baseline and were omitted.

**Model adequacy and diagnostics**

The model was able to retrieve most of the parameters used to generate the in-silico database. However, the 95% credible intervals for $\lambda_{2}^{U}$, $\lambda_{2}^{W}$, $\varepsilon_{FN}^{W}$, $\varepsilon_{FP}^{W}$ and $\varepsilon_{FN}^{U}$ are not included within the 95% credible interval. Results of the model adequacy and diagnostics are shown in Figures S4-S8. The LOOICs of the models indicated that both in the baseline dataset and the dataset in which the cut-off was set at 10, the model that considers cross-reaction performed better (Table S4).

**Figure S4.** Comparison of the model predicted titer responses (model) and the observed titer response (data) for Usutu virus (USUV) and West Nile virus (WNV).

**Figure S5.** Proportion concordant and 95% credible interval of replicates for USUV (A) and WNV (B). The x-axis shows the lowest titer value of the replicate.

**Figure S6.** Posterior distribution (median and 95% credible interval) of the model parameters obtained by fitting the baseline model to the in-silico data. The gray dots are the in-silico parameters used to generate the dataset and the pink interval is the 95% credible interval obtained by fitting the data.

**Table S5.** Model fitness comparison using LOOIC in four different models used in the sensitivity analysis.

|  | Cross-reactivity | No cross-reactivity |
| --- | --- | --- |
| USUV cut-off at 10, WNV cut-off at 30 | 3067.5* (Baseline) | 3212.5 (Model 2) |
| USUV and WNV cut-off at 10 | 3526.2* (Model 1) | 3661.8 (Model 3) |

*best performing model

**Figure S7.** Comparison of prior and posterior distribution for model parameters.

**Figure S8.** Traceplots for all parameters across 4 independent MCMC chains (10000 post-warmup iterations each). Panels show parameters and colors distinguish chains 1–4.

**Table S6.** Posterior summary statistics for all model parameters estimated via sampling (4 chains x 10,000 post-warmup iterations). mean: posterior mean of the parameter across all retained samples; serves as the point estimate; sd: posterior standard deviation; quantifies uncertainty around the mean estimate; n_eff: effective sample size; Rhat: Gelman–Rubin potential scale reduction factor; compares within-chain to between-chain variance. Values at or near 1.00 indicate that all chains have converged to the same distribution.

| **Parameter** | **mean** | **sd** | **n_eff** | **Rhat** |
| --- | --- | --- | --- | --- |
| $p^{W\to U}$ | 0.5183 | 0.1118 | 3738 | 1.0017 |
| $p^{U\to W}$ | 0.6159 | 0.101 | 37102 | 1.0016 |
| ${prop}^{W\to U}$ | 0.3026 | 0.2457 | 12142 | 1.0001 |
| ${prop}^{U\to W}$ | 0.6826 | 0.1954 | 5717 | 1.0012 |
| $\sigma^{W}$ | 0.2492 | 0.0879 | 7687 | 1.0001 |
| $\sigma^{U}$ | 1.3176 | 0.1521 | 5881 | 1.0009 |
| $\lambda_{1}^{W}$ | 0.3369 | 0.1055 | 4372 | 1.0016 |
| $\lambda_{1}^{U}$ | 0.7731 | 0.1167 | 6144 | 1.001 |
| $\lambda_{2}^{W}$ | 0.5367 | 0.1035 | 7706 | 1.0001 |
| $\lambda_{2}^{U}$ | 0.3119 | 0.0845 | 5727 | 1.001 |
| $\lambda_{3}^{W}$ | 0.3754 | 0.1282 | 7755 | 1.0012 |
| $\lambda_{3}^{U}$ | 0.4669 | 0.1255 | 7995 | 1.0004 |
| $\lambda_{4}^{W}$ | 0.6434 | 0.1626 | 6050 | 1.0008 |
| $\lambda_{4}^{U}$ | 0.2969 | 0.1399 | 4384 | 1.0011 |
| $f^{W}$ | 0.4541 | 0.1433 | 7073 | 1.0004 |
| $f^{U}$ | 0.1405 | 0.0912 | 6909 | 1.0001 |
| $\varepsilon^{U}$ | 0.2598 | 0.0289 | 11524 | 1.0001 |
| $\varepsilon^{W}$ | 0.2357 | 0.0343 | 9326 | 1.0002 |
| $\varepsilon_{FP}^{U}$ | 0.0212 | 0.0072 | 10985 | 1.0001 |
| $\varepsilon_{FP}^{W}$ | 0.0919 | 0.0186 | 9967 | 1.0002 |
| $\varepsilon_{FN}^{U}$ | 0.0359 | 0.0224 | 7090 | 1 |
| $\varepsilon_{FN}^{W}$ | 0.104 | 0.026 | 9692 | 1.0003 |
